# Quantitative detection of sleep apnea in adults using inertial measurement unit embedded in wristwatch wearable devices

**DOI:** 10.1038/s41598-024-54817-z

**Published:** 2024-02-19

**Authors:** Junichiro Hayano, Mine Adachi, Fumihiko Sasaki, Emi Yuda

**Affiliations:** 1 Heart Beat Science Lab, Inc., Sendai, Japan; 2 Emeritus Processor, Nagoya City University, Nagoya, Japan; 3 Takaoka Clinic, Nagoya, Japan; 4 Graduate School of Information Sciences, Tohoku University, Sendai, Japan

**Keywords:** Data processing, Respiration, Diagnostic markers

## Abstract

Sleep apnea (SA) is associated with risk of cardiovascular disease, cognitive decline, and accidents due to sleepiness, yet the majority (over 80%) of patients remain undiagnosed. Inertial measurement units (IMUs) are built into modern wearable devices and are capable of long-term continuous measurement with low power consumption. We examined if SA can be detected by an IMU embedded in a wristwatch device. In 122 adults who underwent polysomnography (PSG) examinations, triaxial acceleration and triaxial gyro signals from the IMU were recorded during the PSG. Subjects were divided into a training group and a test groups (both *n* = 61). In the training group, an algorithm was developed to extract signals in the respiratory frequency band (0.13–0.70 Hz) and detect respiratory events as transient (10–90 s) decreases in amplitude. The respiratory event frequency estimated by the algorithm correlated with the apnea–hypopnea index (AHI) of the PSG with *r* = 0.84 in the test group. With the cutoff values determined in the training group, moderate-to-severe SA (AHI ≥ 15) was identified with 85% accuracy and severe SA (AHI ≥ 30) with 89% accuracy in the test group. SA can be quantitatively detected by the IMU embedded in wristwatch wearable devices in adults with suspected SA.

## Introduction

Sleep apnea (SA) is characterized by repeated episodes of apnea and hypopnea due to upper airway collapse and obstruction or cessation of central respiratory drive during sleep associated with subsequent arousal from sleep. SA is a common health problem affecting 10–30% of adults^1,2^, but the consequences are serious^3^. For instance, poor sleep quality due to SA is associated with daytime sleepiness that reduces work performance and cognitive ability and increases the risk of traffic accidents^4–6^. Recurrent nocturnal hypoxia, hypercapnia, and sustained sympathetic activation caused by SA^7^ are associated with increased risks of cognitive dysfunction^8^, systemic hypertension^9^, atrial fibrillation^10,11^, stroke^12^, and nocturnal sudden cardiac death^13^. Polysomnography (PSG) is the gold-standard method for the detection and diagnosis of SA in adults, but there are practical limitations due to the number of PSG testing facilities and testing costs. To overcome this limitation, a number of home portable devices have been proposed for out-of-clinic sleep testing (OCST)^14^ with acceptable agreement with PSG results^15,16^. However, patients with SA, even if they have symptoms such as insomnia or daytime sleepiness, are unlikely to be examined with those SA testing devices, unless they suspect that their symptoms may be caused by SA. In fact, the majority (over 80%) of SA patients still remains undiagnosed^6^ and potential patients with SA are considered a major burden of the healthcare system^3^. There is a need to develop screening methods for SA that have the opportunity to be accessed by more people, including those who may be unaware that they have SA or unaware of the need for SA testing.

Inertial measurement units (IMUs) are embedded in most modern wearable devices and enable continuous measurement of triaxial acceleration and triaxial gyro signals for long period of time with low power consumption. If SA can be detected with IMUs, especially those built into widespread wristwatch-type wearable devices, it is expected that the probability of detecting potential patients will increase. In a previous study, we reported that SA can be detected quantitatively by the analysis of pulse rate data from a wristwatch device’s photoplethysmography (PPG) sensor during sleep^17^, but the PPG sensor requires frequent (usually once a day) battery charging because it consumes more power than IMUs. Additionally, the detection of SA by pulse rate relies on the faithful coupling of SA events and heart rate (cyclic variation of heart rate)^18,19^, which could be impaired by cardiac and neural comorbidity ^20^. In this study, we examined the possibility of detecting SA using the output signal of an IMU embedded in a wristwatch wearable device. This is the first study to show that SA can be detected by an IMU worn on the wrist.

## Methods

### Ethics approval and consent to participate

All procedures were performed in accordance with the Regulations Concerning the Conduct of Life Science and Medical Research Involving Human Subjects at Tohoku University, Japan, the Ethical Guidelines for Medical Research Involving Human Subjects issued by the Japanese Ministry of Health, Labor and Welfare, and the 1964 Declaration of Helsinki and its subsequent amendments. The study protocol was approved by the Research Ethics Committee of the Center for Data-driven Science and Artificial Intelligence, Tohoku University, Japan (No. 2022-4). All subjects participated in this study gave their written informed consent.

### Subjects

The subjects were patients who underwent overnight PSG from November 2022 to March 2023 at Takaoka Clinic (Nagoya, Japan) for the diagnosis of SA or for the evaluation of SA treatment efficacy. The inclusion criterion was adults of age ≥ 20 years. Subjects were excluded if they had acute illness, infectious disease, or chronic disease exacerbation requiring hospitalization within the last 3 months, or were pregnant or breastfeeding.

### Protocol

Subjects visited the sleep clinic in the evening and slept in a PSG testing chamber equipped with an Embla N7000 PSG amplifier (Natus Neurology Incorporated, Middleton, Wisconsin, USA). During PSG testing, a wristwatch device (mSafety, Sony Network Communications Europe, Malmö, Sweden) was worn on the left wrist, and triaxial acceleration and triaxial gyro signals from a built-in IMU (BMI270, Bosch Sensortec GmbH, Reutlingen, Germany) and pulse wave signals from a built-in PPG sensor were recorded simultaneously with the PSG (the pulse wave signals were not used in this study).

Subjects were randomly allocated into a training group and a test group. Using the data from the training group, we developed algorithms to identify SA episodes, constructed regression models to estimate SA severity, and determined the optimal cutoff values for classifying the severity. Using the data from the test group, we evaluated the prediction and classification performance of the algorithms. The measurements were conducted under identical condition between the training and test groups.

### Measurements

The PSG was recorded overnight with the standard PSG montages consisting of F4-M1, F3-M2, C4-M1, C3-M2, O2-M1, and O1-M2 electroencephalograms, left and right electrooculograms, a submental electromyogram, a nasal pressure cannula, oronasal airflows, left and right tibial electromyograms, thoracoabdominal inductance plethysmograms, pulse oxy-metric arterial blood oxygen saturation (SpO2), a neck microphone, body position sensors, and a modified lead II ECG.

The PSG recoding was analyzed offline with a sleep diagnostic software (Remlogic version 3.4.1, Natus Medial Incorporated, Middleton, Wisconsin, USA) and the results of automated analysis were reviewed and edited by expert sleep technicians (Certified Sleep Medicine Examiner by the Japan Sleep Society). Sleep stages and respiratory events were scored according to the American Association of Sleep Medicine (AASM) Manual for the Scoring of Sleep and Associated Events, Version 2.5^21^. The average hourly frequencies of apneic episodes, hypopneic episodes, and the combination were defined as apneic index, hypopneic index, and apnea–hypopnea index (AHI), respectively. The average hourly frequencies of apneic episodes were also measured by the types (obstructive, central, and mixed). AHI was calculated both using the total recording time (TRT) as the denominator (AHI_TRT_) and total sleep time (TST) as the denominator (AHI_TST_). The AHI_TRT_ was used as the reference for the development of the algorithm for detection of respiratory events by IMU signals. The AHI_TST_ was used to determine the severity class of SA in the subjects: subjects with an AHI_TST_ between 15 and 30 as having moderate SA and those with AHI_TST_ ≥ 30 as having severe SA.

The acceleration signal of each axis was recorded at a sampling frequency of 32 Hz and a resolution of 0.061 mG per least significant bit (± 2.0 G at 16 bit) and the gyro signal of each axis was at a sampling frequency of 32 Hz and a resolution of 0.0076 degree per second (dps) per least significant bit (± 250 dps at 16 bit). Acceleration, gyro, and PPG signals were transferred from the wristwatch device to a secure cloud storage via the device's built-in long-term-evolution-for-machine (LTE-M) communication function for constant network connectivity.

### Data analysis

**Figure 1:**
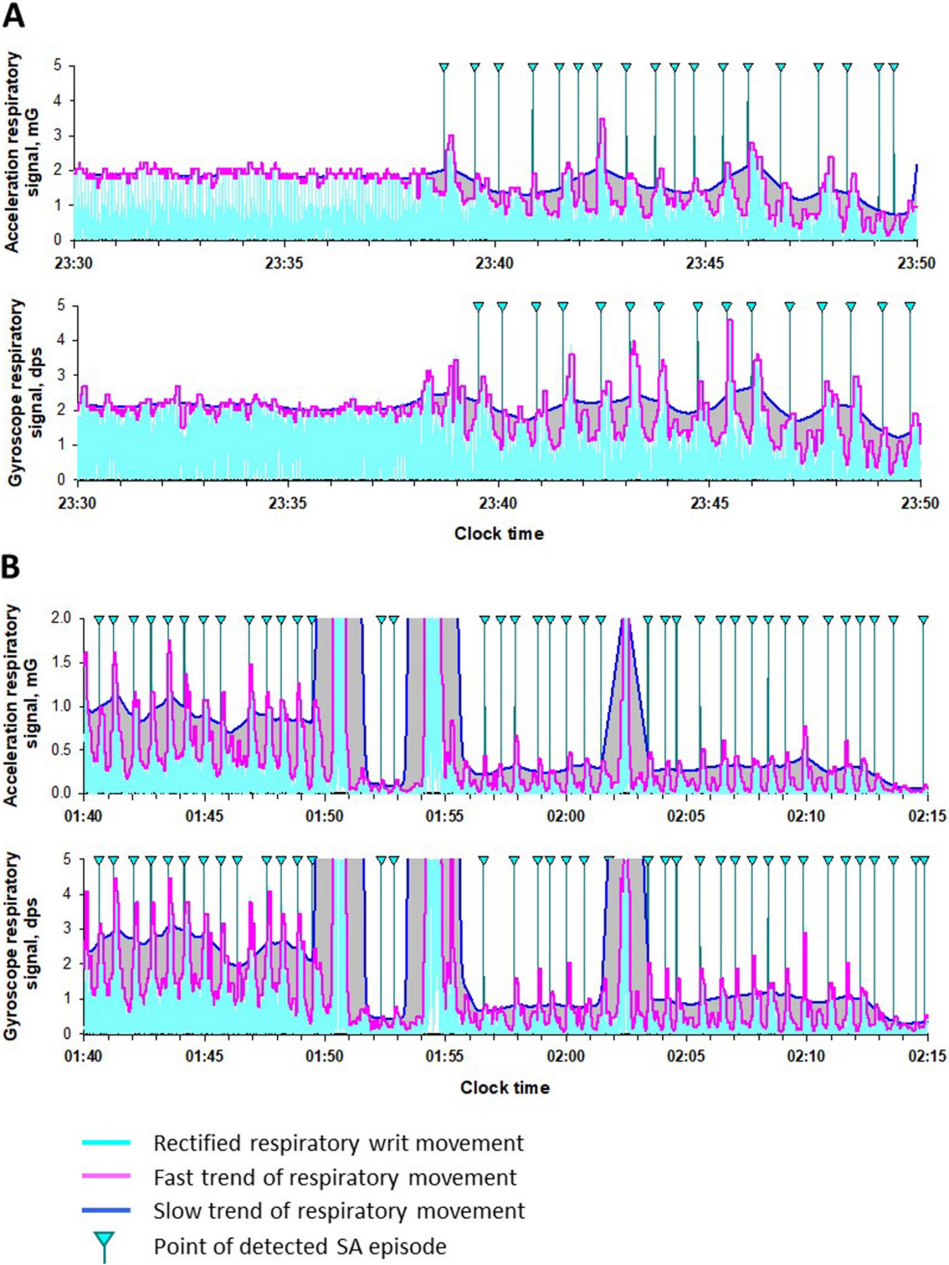
Detection of sleep apnea–hypopnea by acceleration and gyro signals from inertial measurement unit (IMU). (**A**) Rectified respiratory wrist movement (cyan) derived from acceleration (upper row) and gyro (lower row) signals during the transition from normal breathing to a train of sleep apnea (SA) in a representative subject with sever SA. (**B**) Rectified respiratory wrist waveforms (cyan) from acceleration (upper panel) and gyro (lower panel) signals with varying signal intensities during successive episodes of sleep apnea in the same subject. In all panels, blue and magenta lines indicate slow and fast trends, respectively, in the upper envelope of respiratory wrist movement. A drop in the fast trend was determined to be an SA episode if the area bounded by the fast and slow trends was greater than the threshold percentage of area under the slow trend curve and was between 10 and 90 s in the duration. Vertical lines with cyan triangles indicate the points at which an SA episode was detected.

The acceleration signals and gyro signals were analyzed in the same way but separately. For both signals, the entire length (equivalent to the TRT of PSG) of time series were processed as follows. First, each time series of the three axes (X, Y, and Z) was processed separately with a band-pass filter between 0.13 and 0.70 Hz to extract the respiratory signals. Second, the triaxial respiratory signals were combined into a single scalar that reflects respiratory wrist movement,

$${R}_{t}= \sqrt{{X}_{t}^{2}+{Y}_{t}^{2}+{Z}_{t}^{2}},$$

where *Rt* represents time series of respiratory wrist movement and *X*_t_, *Y*_t_ , and *Z*_t_ indicate band-pass filtered X-, Y-, and Z-axis time series, respectively. Third, the time series Rt was band-pass filtered (0.13–0.7 Hz) again to remove the direct-current component and rectified so that the height of upper envelope of the data reflects the amplitude of respiratory wrist movement (Fig. 1). Fourth, considering that the fast trend in the upper envelope reflect the breath-by-breath change in the amplitude of respiratory wrist movement and the slow trend reflects the mean amplitude flattening the SA-induced change in the amplitude, we calculated moving averages of the envelope with averaging window widths of 3 and 30 s for the fast and slow trends, respectively. Fifth, we searched for fast trend drops where the area bounded by the fast and slow trends was greater than a threshold percentage of area under the curve (AUC) of the slow trend, and determined the drop as an SA episode if its length was between 10 and 90 s. Finally, the average number of SA episodes per hour of TRT was calculated as the respiratory event index (REI).

As shown in the first 8 min of Fig. 1A, the fast and slow trends during normal breathing overlap each other and the area bounded by them is small or nonexistence; when SA episode occurs, drop in the fast trend appears and the area bounded by the fast and slow trends increase. The amplitude of respiratory wrist movement could vary with arm and body positions (Fig. 1B). To eliminate the influence of amplitude changes, we evaluated the ratio of the bounded area to the AUC of the slow trend as an index for detecting SA episodes. The threshold for this ratio to detect SA episodes was determined to maximize the multiple correlation coefficient for the AHI_TRT_ regression with acceleration REI and gyro REI in the training group.

### Statistical analysis

The program package of Statistical Analysis System (SAS institute, Cary, NC, USA) was used for statistical analyses. Differences in quantitative and categorical variables between the training and test groups were evaluated by Wilcoxon rank sum test and χ2 test. The relationship between acceleration/gyro REI and AHI_TRT_ was evaluated by linear regression analysis and Pearson's correlation coefficient, and the multivariate predictive power of acceleration and gyro REIs for AHI_TRT_ was evaluated by multiple correlation coefficient using the SAS REG procedure. The AHI_TRT_ estimated by the multiple regression model was used as the respiratory event score (RES). The discriminant performance of the RES between dichotomized subjects by SA severity (cutoff points, AHI_TST_ ≥ 15 and ≥ 30) was evaluated by the AUC of receiver-operating characteristic (ROC) curve. The optimal cutoff values of the RES for the discrimination were determined in the training group and evaluated in the test group with the sensitivity, specificity, accuracy, and positive and negative predictive values (PPV and NPV, respectively). Statistical significance was considered for *P* < 0.05.

## Results

### Subjects’ characteristics

We studied 122 subjects (age range, 21 to 73 years, 14 females) who underwent PSG for diagnostics (*n* = 97, 81%) and evaluation of therapeutic effects (*n* = 25, 19%). Their median (IQR) body mass index was 26.2 (23.9 to 29.7) kg/m^2^. The results of PSG showed that their median (IQR) AHI_TST_ was 17.5 (8.7 to 34.6), that 67 (55%) of them had a moderate-to-severe SA (AHI_TST_ ≥ 15) and 38 (31%) had a severe SA (AHI_TST_ ≥ 15), and that the proportion of obstructive, central, and mixed apnea episodes were 87%, 7%, and 6%, respectively.

For this study, the subjects were randomly divided into the training group (*n* = 61) and the test group (*n* = 61). There was no significant difference in subjects’ characteristics between the two groups (Table 1).

**Table 1:** Subjects’ characteristics in training and test groups.

	Training group (*n* = 61)	Test group (*n* = 61)	*P*^a^
Age, year	49 (41–55)	48 (40–53)	0.4
Female, *n* (%)	6 (10%)	8 (13%)	0.5
BMI, kg/m^2^	26.0 (23.1–29.4)	26.7 (24.1–30.1)	0.3
Purpose of polysomnography			0.3
Diagnosis, *n* (%)	52 (85%)	45 (76%)	
CPAP titration, *n* (%)	4 (7%)	8 (14%)	
Other, *n* (%)	5 (8%)	6 (10%)	
TRT, min	521.5 (508.5–543.0)	515.5 (503.5–531.5)	0.1
Sleep period time, min	507.0 (491.5–528.5)	502.5 (485.0–524.0)	0.2
TST, min	444.0 (363.5–481.5)	440.0 (402.5–475.0)	0.9
Sleep efficiency, %	821.9 (69.8–92.3)	83.1 (76.2–91.4)	0.6
Sleep latency, min	4.5 (2.5–8.5)	6.0 (2.5–11.5)	0.5
Wake after sleep onset, min	67.0 (34.0–116.5)	52.5 (33.5–99.0)	0.4
Sleep stage			
N1, %	24 (17–39)	30 (19–39)	0.3
N2, %	54 (45–61)	52 (42–61)	0.5
N3, %	0 (0–0)	0 (0–0)	0.3
REM, %	19 (14–23)	18 (15–21)	0.2
AHI_TRT_	15.1 (6.6–24.6)	14.3 (7.8–30.5)	0.5
AHI_TST_	17.3 (8.7–34.6)	18.1 (9.1–34.0)	0.7
AI_TST_	3.6 (1.0–13.6)	4.3 (0.9–15.9)	0.6
HI_TST_	10.4 (6.1–16.7)	12.2 (6.5–17.9)	0.5
OAI_TST_	2.3 (0.2–12.9)	3.0 (0.4–15.7)	0.4
CAI_TST_	0.3 (0.1–0.8)	0.3 (0.0–0.7)	0.9
MAI_TST_	0 (0–0.3)	0.1 (0.0–0.4)	0.2
AHI_TST_ ≥ 15	33 (54%)	34 (56%)	0.8
AHI_TST_ ≥ 30	19 (31%)	19 (31%)	1.0

Data are median (IQR) or frequency (%).

*AHI*_*TRT*_ apnea–hypopnea index calculated using total recording time as the denominator, *AHI*_*TST*_ apnea–hypopnea index calculated using total sleep time as the denominator, *AI* apnea index, *BMI* body mass index, *CAI* central apnea index, *CPAP* continuous positive airway pressure, *HI* hypopnea index, *OAI* obstructive apnea index, *MAI* mixed apnea index, *REM* rapid eye movement, *TRT* total recording time, *TST* total sleep time.

^a^Significance of difference (χ^2^ test and Wilcoxon rank sum test for categorical and continuous variables, respectively).

### Development of algorithm to detect SA episodes using IMU signals in the training group

**Figure 2:**
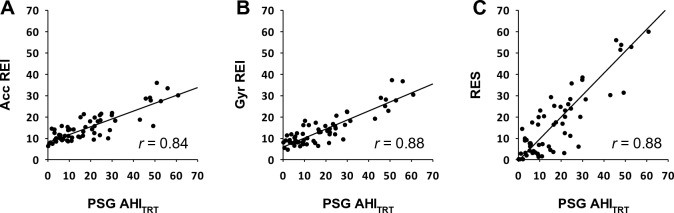
Relationships between apnea–hypopnea index (AHI_TRT_) of polysomnography (PSG) and respiration event index (REI) and respiration event score (RES) obtained from IMU signals in the training group. (A) PSG AHI_TRT_ and REI obtained from acceleration signal (Acc); (B) PSG AHI_TRT_ and REI obtained from gyro signal (Gyr); (C) PSG AHI_TRT_ and RES, an estimate of AHI_TRT_ by combining Acc REI and Gyr REI. *r* = correlation coefficient.

SA detection algorithms for acceleration and gyro signals were developed using data in the training group. The multiple regression of AHI_TRT_ by acceleration and gyro REIs indicated that the greatest squared multiple correlation coefficient (R^2^ = 0.78) was obtained when a threshold of 35% was used to identify drop of the fast trend as an SA episode (Table 2). Therefore, in the following analysis, we decided to use 35% threshold for SA detection. The REI calculated from the acceleration and gyro signals by the SA detection algorithm correlated with the AHI_TRT_ in the training group with correlation coefficients of 0.84 and 0.88, respectively (Fig. 2A, B).

**Table 2:** Correlations between REIs and RES and AHI_TRT_ obtained at different thresholds of SA detection in the training group.

Threshold^a^	Acceleration		Gyro		Multiple regression R^2d^
	REI^b^	*r*^c^	REI^b^	*r*^c^	
10%	49.0 ± 6.8	−0.12	45.9 ± 6.5	0.14	0.27
20%	34.7 ± 5.7	0.5	29.0 ± 7.5	0.74	0.58
25%	26.4 ± 6.1	0.73	22.4 ± 8.0	0.83	0.69
30%	19.6 ± 6.7	0.81	17.4 ± 7.9	0.87	0.77
35%	14.9 ± 6.8	0.84	13.7 ± 7.4	0.88	0.78
40%	11.8 ± 6.6	0.84	10.9 ± 6.8	0.87	0.76
45%	9.6 ± 6.2	0.83	9.0 ± 6.1	0.86	0.74
50%	8.1 ± 5.8	0.83	7.6 ± 5.6	0.85	0.74
60%	6.3 ± 5.1	0.83	6.0 ± 4.9	0.83	0.71

^a^Threshold for detecting respiratory wrist movement reduction based on the ratio of the area bounded by the fast and slow trends to the area under the slow trend (see Fig. 1).

^b^Respiratory event index: number of detected events of respiratory wrist movement reduction by acceleration and gyro signals per hour of TRT.

^c^Correlation coefficient between REI and AHI_TRT_.

^d^Squared multiple correlation coefficient for AHI_TRT_ regression with acceleration REI and gyro REI. A threshold of 35% resulted in the best estimate of AHI_TRT_.

### Development of a respiratory event score (RES) to estimate AHI_TRT_ in the training group

In the training group, REIs obtained from the acceleration and gyro signals were correlated with each other (*r* = 0.93) and there was no significant difference between them (median [IQR], 13.7 [9.8, 18.3] for acceleration and 11.7 [8.1, 16.5] for gyro), but which was larger differed from subject to subject. Therefore, we decided to create a model that combines both to calculate RES as an estimate of AHI_TRT_. Figure 2c shows the relationship between the PSG AHI_TRT_ and RES obtained from the multivariate regression model. The regression model was.

$$RES=1.3 REIacc+1.3 REIgyr-19.2,$$

where REIacc and REIgyr represent the REI obtained from acceleration and gyro signals. ROC analysis of the discrimination performance of RES in identifying subjects with moderate-to-severe SA (AHI_TST_ ≥ 15) and those with severe SA (AHI_TST_ ≥ 30) showed AUC of 0.894 (95%CI, 0.788 to 0.958) and 0.919 (0.820 to 0.973), respectively. The optimal cutoff values for discriminating the subjects with moderate-to-sever SA was RES ≥ 10 (obtained sensitivity, specificity, and accuracy were 85%, 82%, and 84%, respectively) and for those with severe SA was RES ≥ 20 (obtained sensitivity, specificity, and accuracy were 79%, 83%, and 82%, respectively).

### Validation of algorithms and model in the test group

**Figure 3:**
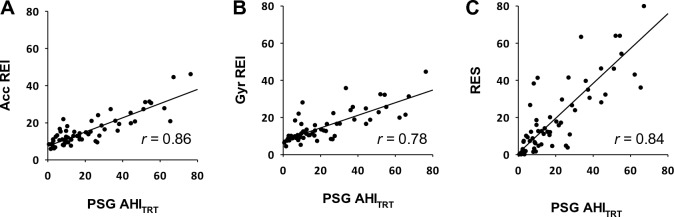
Relationships of (**A**) Acc REI, (**B**) Gyro REI, and (**C**) RES with AHI_TRT_ of PSG in the test group. Acc REI and Gyro REI were calculated from the IMU signals of the test group by the algorithm developed in the training group. The RES was calculated using the regression model developed in the training group. *r* = correlation coefficient.

The REIs of the acceleration and gyro signals calculated by the algorithm were closely correlated (*r* = 0.86 and 0.78, respectively) with AHI_TRT_ in the test group as well (Fig. 3A, B). The RES calculated by the regression model also showed a good correlation (*r* = 0.84) with AHI_TRT_ in the test group (Fig. 3C). Additionally, ROC analysis of the discrimination performance of RES in identifying subjects with moderate-to-severe SA (AHI_TST_ ≥ 15) and those with severe SA (AHI_TST_ ≥ 30) showed AUC of 0.891 (95%CI, 0.785 to 0.956) and 0.921 (0.823 to 0.975), respectively. Tables 3 and 4 show the confusion tables for identification of subjects with different SA severity in the test group using the cutoff values for RES that were determined in the training group. The RES with those cutoff values discriminated moderate-to-severe SA and severe SA with accuracies of 85% and 89%, respectively.

**Table 3:** Confusion table for identification of moderate to severe SA (AHI_TST_ ≥ 15) by RES in the test group.

	AHI_TST_ < 15	AHI_TST_ ≥ 15	
RES < 10^a^	21	3	NPA = 88%
RES ≥ 10^a^	6	31	PPA = 84%
	Specificity = 78%	Sensitivity = 91%	Accuracy = 85%

*NPA* negative predictive accuracy, *PPA* positive predictive accuracy.

^a^Cutoff value of 10 was predetermined in the training group.

**Table 4:** Confusion table for identification of severe SA (AHI_TST_ ≥ 30) by RES in the test group.

	AHI_TST_ < 30	AHI_TST_ ≥ 30	
RES < 20^a^	37	2	NPA = 95%
RES ≥ 20^a^	5	17	PPA = 77%
	Specificity = 88%	Sensitivity = 90%	Accuracy = 89%

^a^Cutoff value of 20 was predetermined in the training group.

Table 5 shows the prevalence of moderate-to-severe SA for multiple levels of RES. If the RES was less than 5, the prevalence of having moderate to severe SA was 17%, but if the RES was 30 or higher, the prevalence was 94%, and if the RES was 40 or higher, the prevalence was 100%. To evaluate the classification performance independent of pretest probability, likelihood ratio was also calculated for each threshold.

**Table 5:** Prevalence of moderate to severe SA (AHI_TST_ ≥ 15) by multiple thresholds of RES in the test group.

RES	AHI_TST_ < 15	AHI_TST_ ≥ 15	Positive LR	Prevalence of SA (%)
< 10	21	3	0.11	13
10–20	4	11	2.18	73
≥ 20	2	20	7.94	91
≥ 30	1	16	12.7	94
≥ 40	0	11	∞	100
Total	27	34		56

*LR* likelihood ratio.

## Discussion

To the best of our knowledge, this is the first study to demonstrate that SA can be quantitatively detected by an IMU embedded in a wearable device worn on the wrist. In this study, we continuously measured the acceleration and gyro signals from an IMU embedded in a wristwatch device during PSG. Using data from half of the subjects, algorithms were developed to identify SA episodes from respiratory wrist movements detected by the acceleration and gyro signals. We also developed a multivariate model to identify SA severity by estimating AHI_TRT_ using SA frequency indices, REIs, provided by the algorithms. In remaining half of the subjects, we tested the accuracies of SA detection and the SA severity classification of the model. We observed that RES, the estimated AHI_TRT_ by the model, correlated well with the AHI_TRT_ (*r* = 0.84), and with the pre-defined cutoff values, identified moderate-to-sever SA with 85% accuracy and severe SA with 89% accuracy.

IMU is widely used for sleep medicine. The obvious utility of IMUs is in determining body positioning and detecting body movements during sleep. IMUs worn on the trunk, legs^22^, and wrists^23^ have been used for assessing sleep quality and detecting specific changes to neurological disorders such as Parkinson's disease^24^ and periodic leg movement^25^. IMUs are also used as sensors to detect seismocardiogram ^26^, and pulse-synchronized jerks in acceleration detected by a wrist-worn IMU are proposed as an indicator for sleep–wake classification^27^. However, none of the previous studies have detected SA using IMU signals recorded at the wrist.

In the present study, we found that respiratory movement can be detected by an IMU at the wrist. The resolutions of the acceleration and gyro signals of the IMU were 0.061 mG and 0.0076 dps, respectively, and the observed respiratory wrist movements had amplitudes 10 to 1000 times greater than those. The amplitude of respiratory wrist movement, however, showed frequent and large fluctuations, possibly due to the changes in body posture and the arm position, making the SA detection dependent on a decrease in absolute respiratory amplitude impossible. To overcome this problem, we introduced an algorithm that uses a fast trend reflecting the breath-to-breath changes in respiratory amplitude and a slow trend flattening the amplitude changes due to SA. SA was detected by a local and relative increase in the area bounded by the fast and slow trends above the threshold percentage of the AUC of slow trend. The threshold percentage was determined to maximize the multivariate correlation between the frequency of detected SA episodes and PSG AHI_TRT_.

Three points need to be mentioned about the methods of data analysis of the present study. First, the acceleration and gyro signals were analyzed separately and SA episodes were detected from each signal. The frequencies of detected SA, REIs, were similar and correlated between acceleration and gyro signals. This suggests that respiration causes both linear movements and rotational movements in the wrist. However, which was larger differed from subject to subject. Therefore, we developed a model that combines both to calculate an estimate of AHI_TRT_, i.e., RES. Given the considerable difference in the correlation between REI and AHI_TRT_ between the acceleration and gyro signals in the test group (Fig. 3), this method, which does not prioritize one over the other, seems effective. Second, while AHI_TST_ is used as a measure of SA severity in clinical PSG testing, we optimized the SA detection algorithm using AHI_TRT_. This was because REI was calculated using TRT as the denominator. On the other hand, AHI_TST_ was used for classifying SA severity by PSG. Consequently, the cutoff values of RES were lower than those of AHI_TST_ for the SA severity classification. We believe this defined RES cutoff value is practical because it can be used with wearable sensors that do not allow for accurate TST estimation. Finally, a strength of this study is that the development of the SA detection algorithms, the determination of the optimal detection threshold, the creation of the model to estimate AHI_TRT_, and the definition of cutoff values were all done in the training group and validated in the independent test group. This framework ensures the reliability of the study results, at least in similar populations.

The Clinical Practice Guideline for Diagnostic Testing for Adult Obstructive Sleep Apnea of AASM^14^ presented the classification performance of six types of Type 3 home SA testing devices in high-risk populations. The reported results indicated sensitivity ranging from 62 to 94%, specificity ranging from 25 to 97%, and accuracy ranging from 65 to 91%. Comparatively, the classification metrics of the present method in the test group were 78%, 91%, and 85%, respectively. Notably, this method measures only two parameters (acceleration and gyro signals), yet it demonstrates favorable performance.

The present findings, demonstrating that SA can be detected by an IMU embedded in a wristwatch device with comparable accuracy to Type 3 devices, open up two possibilities. First, the use of common wristwatch devices for SA screening is to increase the likelihood of detecting SA, especially in those who do not recognize the need for specialized equipment or access to laboratories for SA detection. Given that the majority of SA patients (over 80%) are undiagnosed^6^ and the serious impact of SA on patients’ quality of life and social and health economics^3^, increasing SA screening opportunities is an important social need.

Second, the SA detection capability of the IMU may lead to the development of inexpensive, easy-to-use sleep hygiene devices. IMUs consumes less power than PPG sensors, so they require less frequent battery recharging, and there are fewer measurement failures due to sensor detachment from the skin caused by poor fit or intrusion of ambient light, which sometimes occur with PPG. Analysis of pulse data from PPG sensors has also been proposed as a method for quantitative detection of SA^17^, but this method depends on faithful coupling of SA events to heart rate^18,19^, which can be compromised by cardiac and neurological comorbidity^20^.

A limitation of this study is that the algorithm and model were developed in subjects with suspected SA (54% pretest SA probability, odds = 1.27). If the results of this study were applied to the general population with low pretest probability, similar sensitivity and specificity may not be obtained. To predict the results when applied to populations with different prior probabilities, we also calculated positive likelihood ratio for each threshold value of RES (Table 5). Applying the results of the present study to a population with a pretest SA probability of 10%, the expected prevalence of SA is 1%, 20%, 47%, 59%, and ∼100% when RES is < 10, 10–20, ≥ 20, ≥ 30, and ≥ 40, respectively.

## Conclusions

Acceleration and gyro signals measured during sleep by an IMU embedded in a wrist-worn wristwatch device can be used to detecting SA episodes and estimating SA severity in adults with suspected SA.

## Data Availability

The datasets generated during and/or analyzed during the current study are available from the corresponding author on reasonable request.
